# Supplementary material for: Machine Learning Accelerates Crystallization for Structure Determination
Source: Angew Chem Int Ed Engl. 2026 May 4;65(25):e1218503. doi: 10.1002/anie.1218503 (PMC13266958; doi:10.1002/anie.1218503)

checkCIF/PLATON report

Structure factors have been supplied for datablock(s) lcz019

THIS REPORT IS FOR GUIDANCE ONLY. IF USED AS PART OF A REVIEW PROCEDURE FOR PUBLICATION, IT SHOULD NOT REPLACE THE EXPERTISE OF AN EXPERIENCED CRYSTALLOGRAPHIC REFEREE.

No syntax errors found. CIF dictionary Interpreting this report

Datablock: lcz019

Bond precision:	C-C = 0.0170 A		Wavelength=1.54184
Cell:	a=16.7884(3)	b=16.1378(3)	c=25.0667(4)
	alpha=90	beta=90	gamma=90
Temperature:	100 K		

	Calculated	Reported
Volume	6791.3(2)	6791.3(2)
Space group	P b c a	P b c a
Hall group	-P 2ac 2ab	-P 2ac 2ab
Moiety formula	C25 H15 Ag3 F18 N6 O3	C15 H3 Ag3 F18 N6, C10 H12 O3
Sum formula	C25 H15 Ag3 F18 N6 O3	C25 H15 Ag3 F18 N6 O3
Mr	1113.04	1113.04
Dx,g cm-3	2.177	2.177
Z	8	8
Mu (mm-1)	15.051	15.051
F000	4272.0	4272.0
F000'	4292.85	
h,k,lmax	21,20,31	21,19,31
Nref	7308	7032
Tmin,Tmax	0.205,0.222	0.017,1.000
Tmin'	0.039	

Correction method= # Reported T Limits: Tmin=0.017 Tmax=1.000
AbsCorr = MULTI-SCAN

Data completeness= 0.962   Theta(max)= 78.493

R(reflections)= 0.0756(5857)   wR2(reflections)= 0.1956(7032)

S = 1.151   Npar= 499

The following ALERTS were generated. Each ALERT has the format
test-name_ALERT_alert-type_alert-level.
Click on the hyperlinks for more details of the test.

Alert level C

```
PLAT042_ALERT_1_C Calc. and Reported MoietyFormula Strings  Differ     Please Check
              Calc: C25 H15 Ag3 F18 N6 O3
              Rep.: C15 H3 Ag3 F18 N6, C10 H12 O3
PLAT094_ALERT_2_C Ratio of Maximum / Minimum Residual Density ....       2.31 Report
PLAT213_ALERT_2_C Atom F01H            has ADP max/min Ratio .....        3.6 prolat
PLAT220_ALERT_2_C NonSolvent   Resd 1  F   Ueq(max)/Ueq(min) Range        3.3 Ratio
PLAT234_ALERT_4_C Large Hirshfeld Difference F01H     --C01J     .       0.20 Ang.
PLAT234_ALERT_4_C Large Hirshfeld Difference C01B     --C01D     .       0.16 Ang.
PLAT342_ALERT_3_C Low Bond Precision on  C-C Bonds ..............    0.01695 Ang.
PLAT767_ALERT_4_C INS Embedded LIST 6 Instruction Should be LIST 4     Please Check
PLAT906_ALERT_3_C Large K Value in the Analysis of Variance ......      9.513 Check
PLAT906_ALERT_3_C Large K Value in the Analysis of Variance ......      2.571 Check
PLAT911_ALERT_3_C Missing FCF Refl Between Thmin & STh/L=    0.600         28 Report
                2 19  0,   0  2  1,   1 19  1,   9 17  1,   0 18  2,   1  0  2,
               13 14  7,   0 10  8,   6 16  9,   7 16 10,   7 16 11,   4  4 12,
                9  5 15,  16  3 16,   9  5 18,   9  6 18,  12 10 18,   9  6 19,
               10  6 19,  11  1 20,  15  0 20,   9  4 21,  12  0 22,  12  0 24,
                0  2 25,   6  6 25,   0  4 26,   8  4 26,
PLAT934_ALERT_3_C Number of (Iobs-Icalc)/Sigma(W) > 10 Outliers ..          1 Check
                1 19 11,
PLAT971_ALERT_2_C Check Calcd Resid. Dens.  0.93Ang From Ag01           2.45 eA-3
PLAT971_ALERT_2_C Check Calcd Resid. Dens.  0.87Ang From Ag02           2.33 eA-3
PLAT971_ALERT_2_C Check Calcd Resid. Dens.  0.94Ang From Ag03           2.25 eA-3
PLAT971_ALERT_2_C Check Calcd Resid. Dens.  1.01Ang From Ag01           1.77 eA-3
PLAT971_ALERT_2_C Check Calcd Resid. Dens.  1.05Ang From Ag03           1.76 eA-3
PLAT971_ALERT_2_C Check Calcd Resid. Dens.  0.95Ang From Ag02           1.67 eA-3
```

Alert level G

```
PLAT083_ALERT_2_G SHELXL Second Parameter in WGHT  Unusually Large     121.99 Why ?
PLAT242_ALERT_2_G Low    'MainMol' Ueq as Compared to Neighbors of       C00Q Check
PLAT242_ALERT_2_G Low    'MainMol' Ueq as Compared to Neighbors of       C010 Check
PLAT242_ALERT_2_G Low    'MainMol' Ueq as Compared to Neighbors of       C015 Check
PLAT242_ALERT_2_G Low    'MainMol' Ueq as Compared to Neighbors of       C01G Check
PLAT242_ALERT_2_G Low    'MainMol' Ueq as Compared to Neighbors of       C01I Check
PLAT242_ALERT_2_G Low    'MainMol' Ueq as Compared to Neighbors of       C01J Check
PLAT720_ALERT_4_G Number of Unusual/Non-Standard Labels ..........         70 Note
              Ag01    Ag02    Ag03    F004    F005    F006    F007    F008
              F009    N00A    N00B    F00C    O00D    O00E    F00F    N00G
              N00H    N00I    N00J    F00K    O00L    F00M    F00N    F00O
              F00P    C00Q    F00R    F00S    F00T    F00U    C00V    H00V
              C00W    C00X    C00Y    H00Y    C00Z    C010    C011    H011
```

```
              C012    H012    C013    C014    C015    C016    C017    H017
              C018    C019    H01A    H01B    H01C    C01A    C01B    C01C
              C01D    H01D    H01E    H01F    C01E    H01G    C01F    H01H
              H01I    H01J    C01G    F01H    C01I    C01J
PLAT883_ALERT_1_G Absent Datum for _atom_sites_solution_primary ..     Please Do !
PLAT912_ALERT_4_G Missing # of FCF Reflections Above STh/L=  0.600        218 Note
PLAT941_ALERT_3_G Average HKL Measurement Multiplicity ..........         3.8 Low
PLAT969_ALERT_5_G The 'Henn et al.' R-Factor-gap value ..........       5.983 Note
              Predicted wR2: Based on SigI**2  3.27 or SHELX Weight 16.99
PLAT978_ALERT_2_G Number C-C Bonds with Positive Residual Density.          0 Info
```

```
   0 ALERT level A = Most likely a serious problem - resolve or explain
   0 ALERT level B = A potentially serious problem, consider carefully
  18 ALERT level C = Check. Ensure it is not caused by an omission or oversight
  13 ALERT level G = General information/check it is not something unexpected

   2 ALERT type 1 CIF construction/syntax error, inconsistent or missing data
  17 ALERT type 2 Indicator that the structure model may be wrong or deficient
   6 ALERT type 3 Indicator that the structure quality may be low
   5 ALERT type 4 Improvement, methodology, query or suggestion
   1 ALERT type 5 Informative message, check
```

Validation response form

Please find below a validation response form (VRF) that can be filled in and pasted into your CIF.

```
# start Validation Reply Form
_vrf_PLAT042_lcz019
;
PROBLEM: Calc. and Reported MoietyFormula Strings  Differ     Please Check
RESPONSE: ...
;
_vrf_PLAT094_lcz019
;
PROBLEM: Ratio of Maximum / Minimum Residual Density ....       2.31 Report
RESPONSE: ...
;
_vrf_PLAT213_lcz019
;
PROBLEM: Atom F01H            has ADP max/min Ratio .....        3.6 prolat
RESPONSE: ...
;
_vrf_PLAT220_lcz019
;
PROBLEM: NonSolvent   Resd 1  F   Ueq(max)/Ueq(min) Range        3.3 Ratio
RESPONSE: ...
;
_vrf_PLAT234_lcz019
;
PROBLEM: Large Hirshfeld Difference F01H     --C01J     .       0.20 Ang.
RESPONSE: ...
;
_vrf_PLAT342_lcz019
;
PROBLEM: Low Bond Precision on  C-C Bonds ..............    0.01695 Ang.
```

```
RESPONSE: ...
;
_vrf_PLAT767_lcz019
;
PROBLEM: INS Embedded LIST 6 Instruction Should be LIST 4     Please Check
RESPONSE: ...
;
_vrf_PLAT906_lcz019
;
PROBLEM: Large K Value in the Analysis of Variance ......      9.513 Check
RESPONSE: ...
;
_vrf_PLAT911_lcz019
;
PROBLEM: Missing FCF Refl Between Thmin & STh/L=    0.600         28 Report
RESPONSE: ...
;
_vrf_PLAT934_lcz019
;
PROBLEM: Number of (Iobs-Icalc)/Sigma(W) > 10 Outliers ..          1 Check
RESPONSE: ...
;
_vrf_PLAT971_lcz019
;
PROBLEM: Check Calcd Resid. Dens.  0.93Ang From Ag01           2.45 eA-3
RESPONSE: ...
;
# end Validation Reply Form
```

It is advisable to attempt to resolve as many as possible of the alerts in all categories. Often the minor alerts point to easily fixed oversights, errors and omissions in your CIF or refinement strategy, so attention to these fine details can be worthwhile. It is up to the individual to critically assess their own results and, if necessary, seek expert advice.

PLATON version of 17/09/2025; check.def file version of 17/09/2025

---

duplicate check

No duplication found

Datablock lcz019 - ellipsoid plot

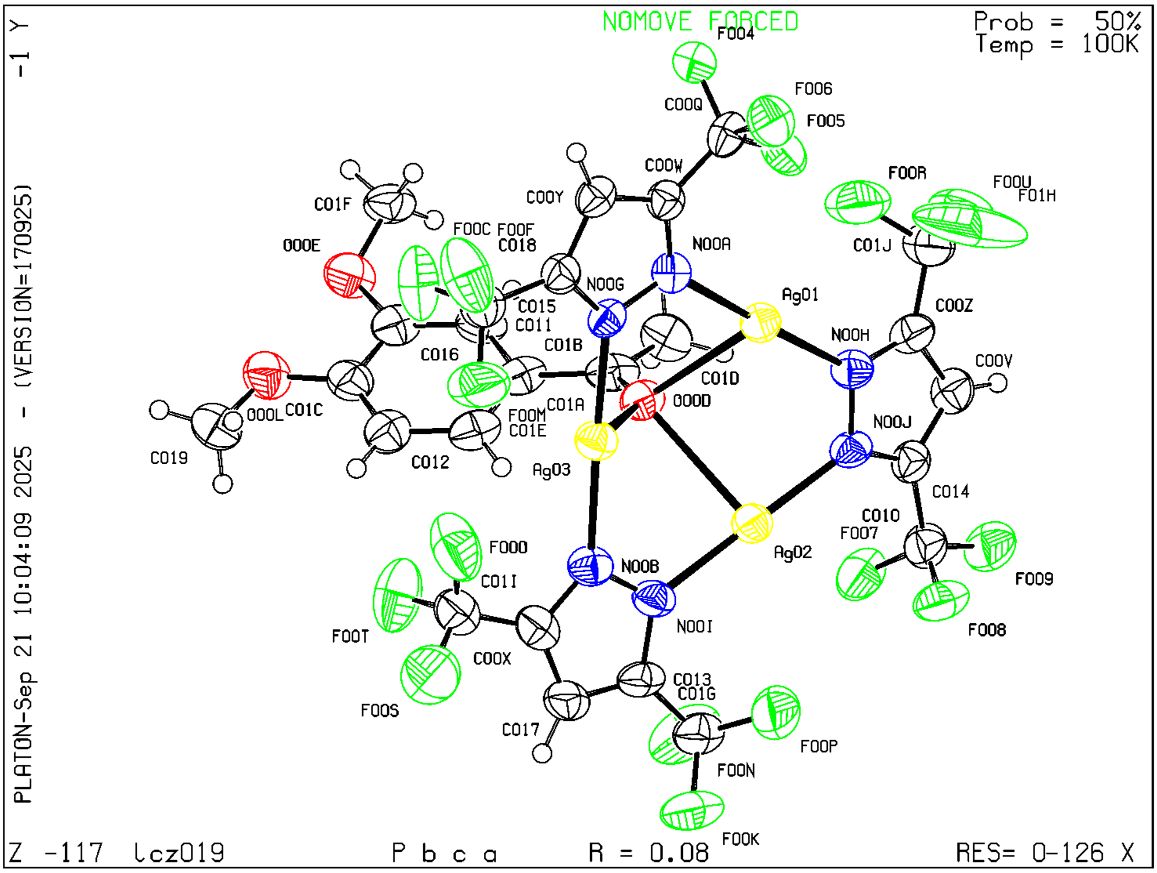

Supplement: Supplementary file 2 — Supporting File 2: anie72462‐sup‐0002‐DataFile.zip. [file ANIE-65-e1218503-s002.zip › supplementary_materials_crystallographic_data submit.zip!/cif_and_checkcif(1-114)/Ag3Pz3_19/Ag3Pz3_19_checkcif.pdf]
